# Infantile juvenile xanthogranuloma of the chest wall mimicking mesenchymal hamartoma: report of a case

**DOI:** 10.1007/s00383-012-3137-z

**Published:** 2012-08-03

**Authors:** Daisuke Masui, Suguru Fukahori, Kimio Asagiri, Yoshiaki Tanaka, Shinji Ishii, Shinichiro Kojima, Motomu Yoshida, Naoko Komatsuzaki, Ken Tanikawa, Masayoshi Kage, Shuji Nagata, Minoru Yagi

**Affiliations:** 1 Department of Pediatric Surgery, Kurume University School of Medicine, 67 Asahi-machi, Kurume, Fukuoka, 830-0011 Japan; 2 Department of Pathology, Kurume University School of Medicine, 67 Asahi-machi, Kurume, Fukuoka, 830-0011 Japan; 3 Department of Radiology, Kurume University School of Medicine, 67 Asahi-machi, Kurume, Fukuoka, 830-0011 Japan

**Keywords:** Chest wall tumor, Juvenile xanthogranuloma, Mesenchymal hamartoma, Langerhans cell Hystiocytosis

## Abstract

Juvenile xanthogranuloma (JXG) is essentially a benign neoplasm arising from any site on the body; however, there has so far been only one report of JXG located on the chest wall involving a rib. This report presents a rare case finally diagnosed as JXG based on histopathological and immunohistochemical examinations.

## Introduction

Pediatric chest wall tumors are relatively rare and include a variety of diseases. Malignant chest wall tumors, especially Ewing sarcoma and the primitive neuroectodermal family, are found more frequently than benign lesions. Therefore, a histopathological examination is often required in case the definite diagnosis is not revealed during clinical course. This report presents a rare case finally diagnosed as juvenile xanthogranuloma (JXG) based on a histopathological examination, although mesenchymal hamartoma (MH) was suspected from the clinical course and the radiological examinations.

## Case report

**Fig. 1:**
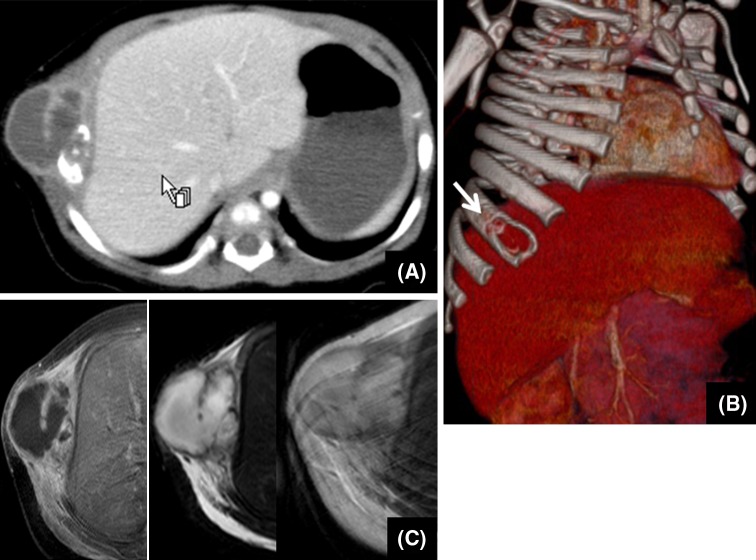
Chest CT demonstrated a multicystic mass, sized 28 × 27 mm and enhanced homogeneously **(a)**. A three-dimensional CT image clarified an osteolytic finding (*←* *arrow*) (**b)**. A MRI revealed an enhanced thickened septum in the tumor **(c)**

A 3-month-old female, with no complications at birth, was referred to our hospital for an enlarging right chest wall mass that had not responded to antibiotic therapy. A physical examination demonstrated an approximately 5 cm subcutaneous mass on the right chest wall, accompanied by skin redness and tenderness. Laboratory examinations demonstrated leukocytosis (11,000/μl) and high levels of CRP (4.8 mg/dl), whereas tumor markers showed levels within the normal range. A chest radiograph showed an increased radiolucent lesion on the right 8th rib. Chest computed tomography (CT) revealed a multicystic mass (28 × 27 mm) with enhanced homogeneously (Fig. 1a). Three-dimensional CT image indicated an osteolytic finding (Fig. 1b). Magnetic resonance imaging (MRI) revealed an enhanced thickened septum in the tumor (Fig. 1c). These findings suggested MH. However, malignant neoplasms, such as Ewing sarcoma, neuroblastoma, fibrosarcoma, rhabdomyosarcoma and chondroblastoma, which are also characterized by cystic osteolytic findings, were also considered as alternative diagnoses. The tumor showed self-destruction with the discharge of a serous fluid and it decreased dramatically in size throughout the clinical course. The skin redness and tenderness also gradually disappeared. A benign tumor was strongly suggested by the sudden regression; however, tumor resection was performed to obtain a definite diagnosis. Poorly demarcated fibrous tissue was located subcutaneously, with slight adhesion to the surface of the 8th rib. The tumor was excised with the surrounding tissue.

**Fig. 2:**
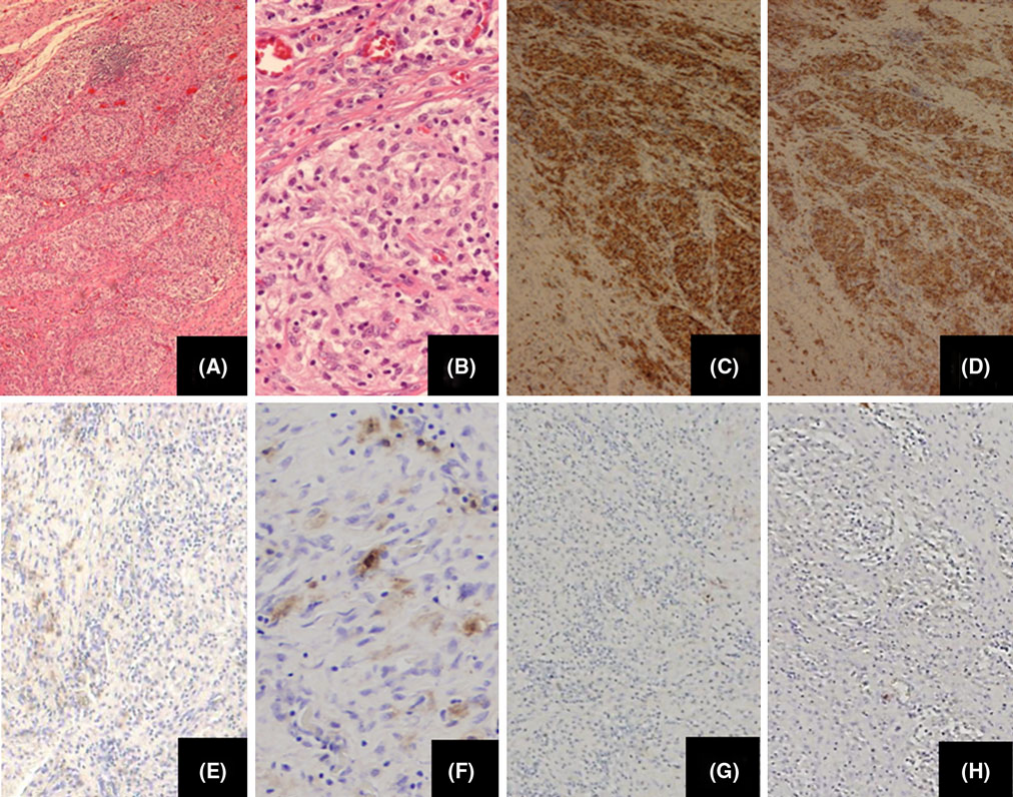
The histological findings showed foamy cytoplasmed cells aggregated sheet-like or lobular-like into the interstitium. The lymphocyte infiltration was observed on the periphery. The cells were composed from histiocyte **(a** H&E, ×40; **b** H&E, ×100**)** In the immunohistochemical examinations, the cells were positive for Kp-1 (**c** immunohistochemistry of Kp-1, ×40), CD163 (**d** immunohistochemistry of CD163, ×40), focally positive for Factor X111a (**e** Immunohistochemistry of Factor X111a, ×40, **f** Immunohistochemistry of Factor X111a, ×100), whereas the cells were negative for S100 (**g** immunohistochemistry of S100, ×40) and CD1a (**g** immunohistochemistry of CD1a, ×40)

The histological findings showed cells with foamy cytoplasm in a sheet-like or lobular-like aggregation in the interstitium. Lymphocyte infiltration was observed on the periphery. The cells were composed of histiocytes (Fig. 2a, b). These findings suggested that histiocytic granulation tissue are accompanied by fibrotic change. There were no atypical cells and findings are consistent with MH. Immunohistochemical investigations have shown that the cells to be positive for Kp-1 (Fig. 2c), CD163 (Fig. 2d), focally positive for Factor 13a (Fig. 2e, f), whereas they were negative for S100 (Fig. 2g), CD1a (Fig. 2h). The histological and immunohistochemical findings indicated that the chest wall tumor was a JXG. The postoperative course was uneventful and there was no evidence of recurrence during a 1-year follow-up at the out-patient clinic.

## Discussion

Pediatric chest wall tumors are relatively rare and only account for approximately 1.8 % of all solid tumors in pediatric patients [1–3]. Benign tumors, such as MH, Langerhans cell histocytosis (LCH), aneurismal bone cyst and osteoma are much rarer than malignant tumors. MH is the most common benign chest wall tumor with an inner cystic component. The rib is the most common site of predilection [1]. Osteolytic finding are frequently observed in malignant tumors such as neuroblastoma, primitive neuroectodermal tumor, hematological tumors and primary soft tissue tumors such as Ewing sarcoma, fibrosarcoma, rhabdomyosarcoma and chondroblastoma. Whereas this is rare in benign tumors, MH demonstrates inner bone destruction and bulging [1]. MH arises from the ribs and forms a subcutaneous mass along the chest wall in the neonatal period or infancy. The vast majority of such cases are located on the chest wall, arise in the ribs and are often noted by swelling of the chest wall or detected incidentally by a chest radiograph [2].

The chest X-ray findings show a large expansive mass that has well-defined sclerotic margins. CT demonstrates a heterogeneous extrapleural tumor mixed with a solitary component and a cystic component filled with fluid characteristic of hemorrhaging. MRI also demonstrates a heterogeneous middle signal intensity mass in both T1 and T2 weighed images [1, 2]. MH contains solid and cystic lesion in which there is proliferation of fibroblastic cells, osteocytes, chondrocytes and hemorrhagic cysts. The differential diagnosis includes chondrosarcoma, aneurysmal bone cyst, chondroma and osteosarcoma. However, these diseases can generally be ruled out without performing immunohistochemistry. Most of the MH demonstrate the spontaneous regression. The present case showed spontaneous tumor regression and an osteolytic finding in the clinical course, which was quite similar to the typical clinical course of MH, although the tumor size was smaller [1–3].

On the other hand, JXG is known as a benign histolytic tumor, which develops within several months of birth and regresses spontaneously. JXG arises from any site of the body and is predominantly located on the surface of the skin, in particular, on the face, extremities and the trunk. In addition, this tumor is sometimes detected in the deep dermis or muscular layer, and has been found in organ tissues [4–7]. A review of 174 JXG cases found that 28 cases (16 %) were solitary subcutaneous or deep soft tissue masses, and only 5 (2.9 %) cases arose from bone lesions. Only three of those cases were centered in the bone and occurred in the temporal-petrous bone in two and the lumbar vertebra in the other [4]. Only one report has so far described a case involving a rib [8]. However, this case did not have any obvious osteolytic findings as was observed in the current case.

The prognosis of JXG is essentially good. However, despite its self-regressive nature, a tumor resection is frequently conducted to obtain a definite diagnosis in most cases even if located on the skin. Histiocytic nodular infiltration in all the layers of the dermis, accompanied by foamy cells and Touton multinucleated giant cells, are also observed [8].

An osteolytic finding due to JXG is extremely rare, therefore, it is hard to obtain a definitive diagnosis by only radiological examinations. Surgical excision was required for the definite diagnosis in the current case, to determine the further therapeutic course [4]. LCH should be differentiated from JXG in histopathological findings [9]. LCH develops commonly in the infant and toddler period. The most common sites of involvement are the bone, lung, central nervous system, liver, thymus, skin and lymph nodes. Up to 90 % of LCH in children occurs in bone [10]. Any bony lesion can be involved in LCH, whereas the skull is the most frequently involved, followed by the pelvis, spine, mandible and ribs [11, 12].There have been several LCH case reports involving the sternum [13]. The X-ray findings demonstrate an osteolytic lesion with relatively sharp margins and a punched out appearance.

The histopathological findings show the proliferation of dendritic cells and also demonstrate giant cells similar to JXG. Therefore, an immunohistochemical examination is useful for further investigation to distinguish these tumors [8, 14]. JXG demonstrates positive in CD68 and factor 13a in immunohistochemical studies, whereas negative staining for CD1a and S-100. On the other hand, LCH shows all positive CD68, CD1a and S-100 [8, 14]. The prognosis of LCH varies from spontaneous regression to cases with poor outcomes, especially with multi-organ involvement. There is no standardized treatment modality for LCH. Solitary bone involvement can often regress spontaneously or after percutaneous needle biopsy. Surgical curettage and direct intralesional injection of methylprednisolone are reported as effective therapies [15, 16].

The clinical difference between JXG and LCH is that the common site: skin JXG versus bone LCH [6, 17]. The percentage of spontaneous regression is higher in JXG than LCH which could affect the prognosis. The present case revealed osteolytic findings in the image studies, which made it difficult for a definitive diagnosis. Finally, histopathological examinations with immunohistochemistry have been reported to be quite useful for distinguishing between JXG and LCH [6, 8].

In conclusion, a high proportion of the pediatric chest wall tumors are generally malignant. It is intolerable for clinicians to observe a growing mass over the long term without a definite diagnosis. Therefore, an incisional biopsy should not be delayed in order to achieve a definitive diagnosis and to initiate a prompt therapeutic course. JXG is essentially a benign neoplasm arising from any site on the body, whereas, there has been only one report of JXG located on the chest wall involving a rib. The present case demonstrated osteolytic findings, therefore, surgical excision was conducted to rule out the presence of malignant neoplasm and an immunohistochemical examination was useful for distinguishing the lesion from LCH. JXG should therefore be considered in the differential diagnosis of a pediatric chest wall tumor.
